# Optimisation of a composite difference metric for prompt error detection in real-time portal dosimetry of simulated volumetric modulated arc therapy

**DOI:** 10.1259/bjr.20201014

**Published:** 2021-03-18

**Authors:** James L Bedford, Ian M Hanson

**Affiliations:** 1 Joint Department of Physics, The Institute of Cancer Research and The Royal Marsden NHS Foundation Trust, London, UK

## Abstract

**Objectives:**

In real-time portal dosimetry, thresholds are set for several measures of difference between predicted and measured images, and signals larger than those thresholds signify an error. The aim of this work is to investigate the use of an additional composite difference metric (CDM) for earlier detection of errors.

**Methods:**

Portal images were predicted for the volumetric modulated arc therapy plans of six prostate patients. Errors in monitor units, aperture opening, aperture position and path length were deliberately introduced into all 180 segments of the treatment plans, and these plans were delivered to a water-equivalent phantom. Four different metrics, consisting of central axis signal, mean image value and two image difference measures, were used to identify errors, and a CDM was added, consisting of a weighted power sum of the individual metrics. To optimise the weights of the CDM and to evaluate the resulting timeliness of error detection, a leave-pair-out strategy was used. For each combination of four patients, the weights of the CDM were determined by an exhaustive search, and the result was evaluated on the remaining two patients.

**Results:**

The median segment index at which the errors were identified was 87 (range 40–130) when using all of the individual metrics separately. Using a CDM as well as multiple separate metrics reduced this to 73 (35–95). The median weighting factors of the four metrics constituting the composite were (0.15, 0.10, 0.15, 0.00). Due to selection of suitable threshold levels, there was only one false positive result in the six patients.

**Conclusion:**

This study shows that, in conjunction with appropriate error thresholds, use of a CDM is able to identify increased image differences around 20% earlier than the separate measures.

**Advances in knowledge:**

This study shows the value of combining difference metrics to allow earlier detection of errors during real-time portal dosimetry for volumetric modulated arc therapy treatment.

## Introduction

Portal dosimetry is a valuable means of ensuring that the dose delivered to the patient during a fraction of radiotherapy is in agreement with that predicted by the treatment planning system.^1–3^ The two principal methods are forward projection,^4,5^ in which portal images are predicted at the time of treatment planning, and then the delivered images are compared with these; and back projection,^6–8^ in which the measured images are projected into the CT scan of the patient and converted into a dose distribution, which is then compared with the planned dose distribution.

The most widespread use of portal dosimetry is for verification of whole fractions of treatment, with analysis being carried out after delivery of the complete fraction.^9^ Often this is the first fraction or closely thereafter, with additional measurements and analyses being carried out when anatomical variations occur, subject to departmental resources.^10–12^ However, real-time or intrafraction portal dosimetry is used to evaluate each fraction of the treatment as it is delivered.^13–16^ This has the advantage that errors can be detected before the whole fraction of treatment has been delivered. With a constant tendency for treatments to become more hypofractionated, either in a stereotactic context^17,18^ or otherwise,^19^ this approach is important, as a whole fraction of treatment accounts for a large proportion of the total dose delivered. The approach also allows for a time-resolved analysis, which gives greater insight into the accuracy of the delivery than when examining the whole.^20,21^

**Figure 1.**
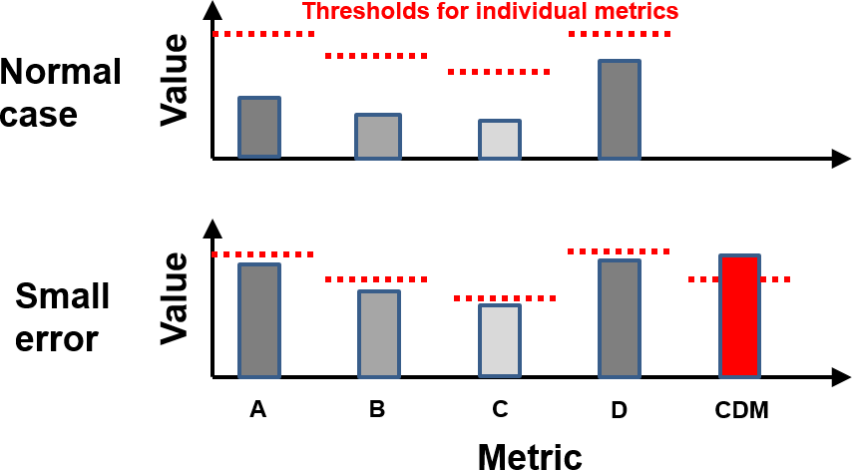
Principle of the CDM. The individual metrics do not respond strongly enough to an error to exceed their respective thresholds, but the composite difference metric detects the error. For a large error, most or all of the metrics exceed their thresholds. CDM, composite difference metric.

The selection of appropriate metrics for optimum sensitivity and specificity of portal dosimetry is the subject of continuing research. Olaciregui-Ruiz et al^22^ investigate a number of different treatment plan metrics and report on their success in identifying errors in a large cohort of volumetric modulated arc therapy (VMAT) patients. Monitoring and analysis software typically follows several metrics simultaneously, using appropriate thresholds for each. However, there may be value in using a single composite difference metric (CDM). This is convenient for monitoring purposes, but the principal rationale is that small but correlated changes in the individual measures may cause the composite metric to indicate an error before any of the individual measures has exceeded its own threshold (Figure 1).

This study investigates a composite difference metric consisting of a weighted power sum of several individual metrics. The method is applied to segment-resolved portal dosimetry for VMAT treatment for prostate. Various errors are deliberately introduced into the treatment plans and the resulting plans are delivered to a water-equivalent phantom. Several metrics are used individually and simultaneously to detect differences between the predicted and measured images. A composite difference metric is proposed, and an optimisation method to determine the optimum metric weights is described. The sensitivity of the method to the various errors is determined, and the number of segments taken during delivery of a treatment fraction for each error to become apparent is used as an indicator of success.

## Methods and materials

### Patients and treatment plans

Treatment plans for six prostate patients were retrospectively used in this study. The patients consented for their images to be used for research purposes. Three planning target volumes (PTVs) were used to create the treatment plans, two based on the prostate alone and the third, outermost volume, based on the prostate plus seminal vesicles.^23,24^ The prescribed dose was 60 Gy in 20 fractions to the most central PTV. The treatment plans consisted of a single 358° gantry arc comprised of 180 segments at a spacing of 2°, with collimator angle 2° throughout. The plans were created using AutoBeam v5.8^25^ using a dose grid of 4 mm × 4 mm × 4 mm resolution and a final calculation and renormalisation was carried out with Pinnacle^3^ (Philips Radiation Oncology Systems, Madison, WI) using a dose grid of 2.5 mm × 2.5 mm × 2.5 mm resolution. The beam energy was 6 MV and the plans were designed for delivery with a VersaHD accelerator (Elekta AB, Stockholm, Sweden).

After inverse planning, AutoBeam was used to recalculate the plan on a water-equivalent phantom of dimensions 300 mm long (G−T direction) × 300 mm wide (A−B direction) × 200 mm high, with the isocentre located at the centre of the phantom. The point dose at the isocentre and the mean dose over a volume corresponding to the 24 mm length and 6 mm diameter of a Farmer 2571 ionisation chamber (Saint Gobain Crystals and Detectors, Reading, UK) were noted for verification purposes.

### Predicted images

The AutoBeam software was used to calculate predicted portal images for each treatment plan. The prediction model projected the dose distribution at the isocentre plane onto the image plane with 512 × 512 pixels of size 0.8 mm × 0.8 mm at a source-panel distance of 1600 mm, giving a pixel size of 0.5 mm at the isocentre plane. The prediction model distinguished between the in-field region of the image panel, defined as the region within the beam’s eye view of the treatment beam, and the out-of-field region, which was the remaining part of the panel. The in-field signal was calculated as the dose at the isocentre plane, corrected to give dose in a water-equivalent medium, and projected to the image plane, allowing for exponential attenuation. The out-of-field signal was calculated as a uniform signal, proportional to the field size and based on a scatter factor as a function of the mean equivalent path length from isocentre plane to detector plane. The union of both in-field and out-of-field regions was then convolved with a pair of Gaussian scatter kernels to allow for in-panel scatter.^5^

Previous work^16,26^ indicated that an accurate prediction model was important. This was because a more accurate prediction led to a lower error threshold, which in turn allowed for greater sensitivity to errors. In the present study, a model of couch attenuation between the isocentre and the image panel was therefore included. This model consisted of an additional radiological path length between the isocentre plane and the image plane, for gantry angles between 300° and 60° through 0°. The additional path length was 8.0 mm, divided by the cosine of the gantry angle so as to allow for oblique passage through the couch. The path length at gantry angle 60° was therefore 16 mm.

A model of gantry and panel sag was also used to account for any movement of the radiation isocentre and image panel. Portal images for a 100 mm × 100 mm field were acquired at all gantry angles at intervals of 10° and the centre of the imaged field and the central image intensity were evaluated. Lateral displacement of the panel and variation in intensity were negligible, but the longitudinal displacement of the panel was found to be significant and predictable. This was similar to the observations of Poludniowski et al^27^. The longitudinal displacement was therefore included in the prediction model as a sinusoidal shift with peak-to-peak amplitude of 4 mm.

Predicted images were produced for each control point of the VMAT arc and stored as a single binary file for each patient, which was subsequently loaded into the AutoDose v. 1.1 software to provide the predictions needed for comparison with real-time images.^16^

### Measured images

The treatment plans were delivered to a Solid Water phantom (Radiation Measurements, Inc., Middleton, WI), with geometry as described above. The dose at the isocentre was verified using a Farmer ionisation chamber, corrected for accelerator output. From the treatment plan for each of the patients, further plans were created by deliberately introducing errors into all 180 segments. The errors consisted of a 4% increase in monitor units, a retraction of 4 mm of all multileaf collimator (MLC) leaves, a shift of 4 mm of all MLC leaves, and introduction of an air space of 20 mm width (left-right direction) × 50 mm height (anteroposterior direction) into the phantom, this latter to simulate rectal gas.^26^ In three of the patients, the magnitudes of the errors were varied to give a 2–10% increase in monitor units in 2% steps, a retraction of 2–10 mm in 2 mm steps of all MLC leaves, a shift of 2–10 mm in 2 mm steps of all MLC leaves, and introduction of an air space of 10–50 mm width in 10 mm steps into the phantom. These systematic variations ensured that the method was able to detect errors of various magnitudes. The correct and erroneous treatment plans were then delivered to the water-equivalent phantom.

Portal images were measured using an iViewGT portal imager (Elekta), which consisted of an amorphous silicon panel (Perkin Elmer, Santa Clara, CA), recording images in JPEG format at a size of 512 × 512 pixels.^28^ The AutoDose software used the gantry angle information stored with each image to bin the images according to treatment plan segment. In a real-time context, the measured images would be compared with the predicted images as the treatment progressed, and categorised according to a traffic light system, but in this study, the images were stored and processed retrospectively, segment by segment. No correction for accelerator output was applied in this process. Both predicted and measured images were rescaled by 10^−4^ to bring them into a range of approximately 0–100, thereby allowing convenient handling.

### Difference metrics

Four metrics were used to identify differences between predicted and measured images: central axis signal (CAS), mean image value (MIV), mean absolute difference as a percentage of maximum predicted image intensity (MDM) and mean absolute difference as a percentage of local predicted image intensity (MDL). The latter three of these metrics were computed for regions where the predicted image had an intensity of greater than 10% of the maximum. A composite difference metric was then added:

(1) $$CDM=\sum_{i=1}^{4}w_{i}m_{i}^{w_{i}}$$

where *m_i_* were the individual metrics and *w_i_* were weighting factors, which took values of between 0 and 1. The first two metrics had dimensions of image intensity and the latter two were percentages, but the rescaling of the image intensities allowed for practical computation of the CDM. The CDM itself was considered as a measure of the presence of an error, analogous to a human operator assessing a variety of measures, with their respective dimensions, and making a decision as to whether there was an error.

This form of metric was found by empirical investigation. Other forms, such as a simple weighted sum or product were found to be less effective, so were not pursued. However, from a theoretical perspective, the usefulness of Equation (1) can be seen from the fact that with *w_i_* less than 1, the CDM takes the form of a sum of functions similar to$y=\sqrt{x}$. These functions increase rapidly at low values of *x* and then plateau. The result is that the CDM has the maximum impact when all of the individual metrics are raised slightly, in contrast to a single metric with a large elevation in value. The weights are equal to the powers to limit the number of combinations of parameter to be searched, while allowing a broad search space.

**Figure 2.**
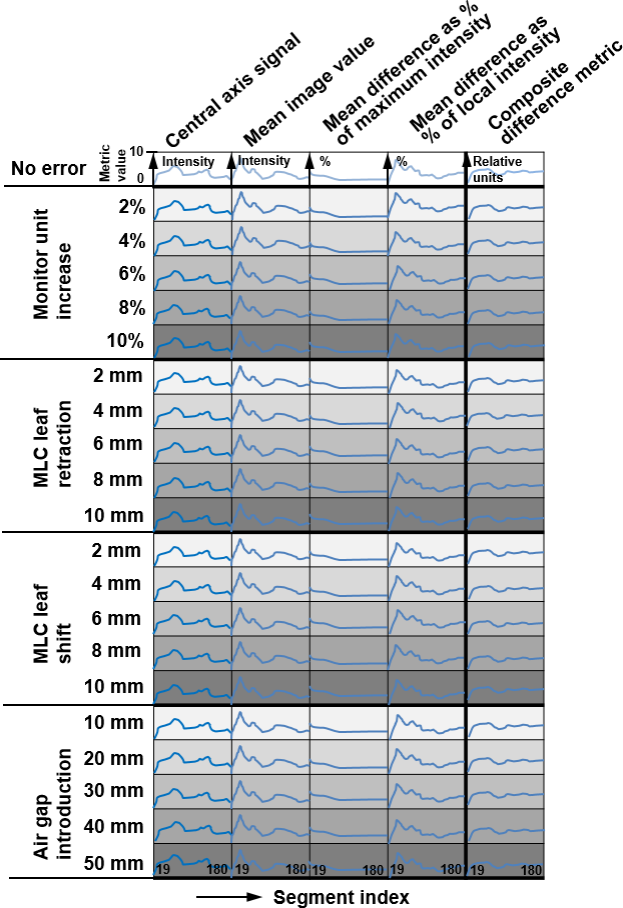
Summary of data used for evaluation of the composite difference metric. Each cell represents a sequence of metric values for segments 19–180 of the treatment plan (shown schematically only). There are four metrics and the composite difference metric. Measurements are available for no error and increasing error of one of four types. MLC, multileaf collimator.

The composite difference metric was calculated for each segment of the treatment plan. The first 10% of segments were not considered as the image signal was unstable in this period. The segments of the plans were approximately evenly weighted, so that the first 10% of segments was expected to deliver approximately 10% of the dose. By removing these segments from the analysis, the potential existed for an error in this part of the plan to remain unnoticed, allowing incorrect dose delivery. Such an error was expected to be much less than the total dose delivered by the initial segments. After the first 10% of segments, a running sum of 10 segments, or “section value”, was used to provide information on each part of the VMAT arc while minimising image fluctuation. The section value for segment *s* consisted of the sum of segments *s* - 9 to *s*. Figure 2 summarises the data used.

### Optimisation of the composite difference metric

Based on the above arrangement of data, an optimisation routine was used to determine the optimal values of the weighting factors in the CDM. In order to ensure that the results were generally applicable and not just optimal for the patients used in the optimisation, a leave-pair-out strategy was used. In the development of prediction models, particularly in artificial intelligence, an optimisation method is commonly used to ensure optimal prediction. When the sample used for optimisation is limited, the possibility exists that the resulting parameters give good prediction for that sample only, and rather less good results in further patients, in which case overfitting is said to occur. Cross-validation is used to avoid this, by selecting an optimisation sample for the fitting and then a validation sample for testing the results. In leave-pair-out cross-validation, each pair of data items is set aside in turn for validation, while the remaining data items are used for optimisation. The interested reader is referred to the more formal descriptions given by Kohavi^29^ and Hastie et al^30^. In the context of this study, the CDM weights for four of the patients were optimised, and the results were then applied to the remaining two patients. For purposes of uniformity, the optimisation set contained two patients with errors of varying magnitude and two patients without (see Measured images), while the test set contained one patient with errors of varying magnitude and one without. This gave nine unique combinations of optimisation set and test set. The process described below was therefore carried out nine times and the median and range of the results recorded for the test patients.

**Figure 3.**
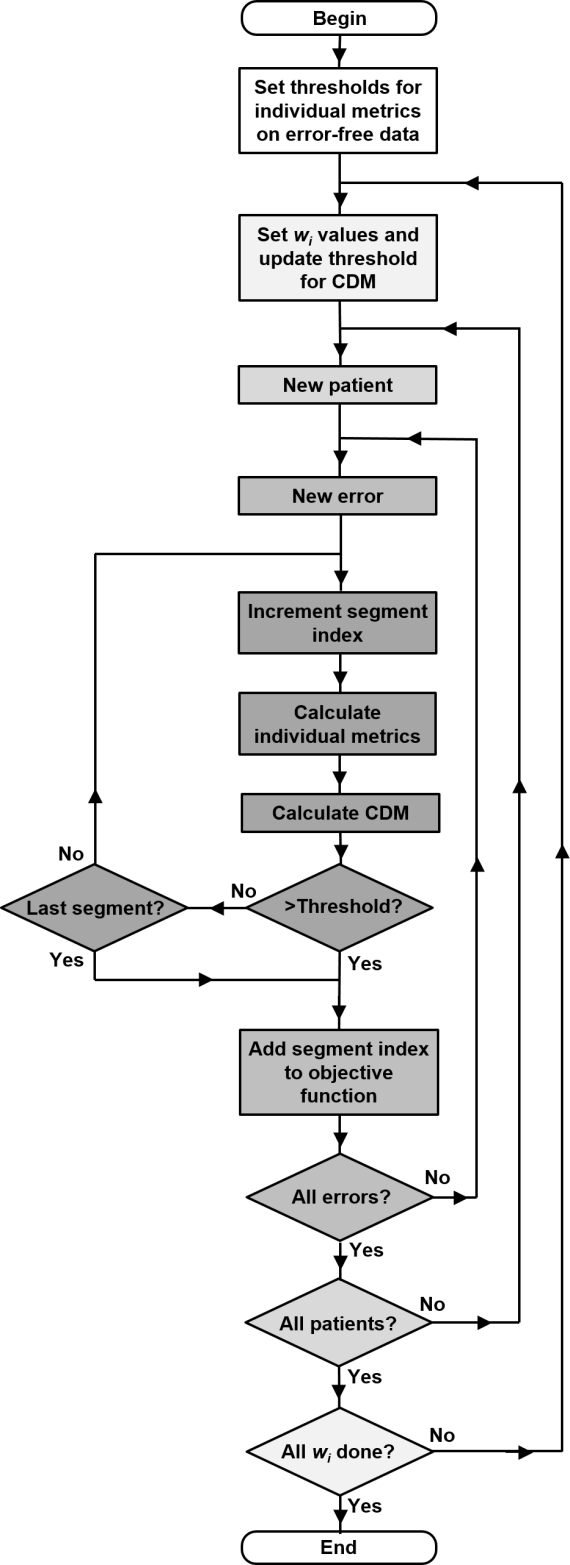
Flow diagram showing the optimisation process for determination of weighting factors in the composite difference metric. The greyscale intensity represents the computational workload. CDM, composite difference metric.

The first step in the optimisation of CDM weights (Figure 3) was to calculate the thresholds for the individual metrics, based on the normal deliveries of the patients (the top row of Figure 2). In order to avoid the possibility of false-positive results occurring when transferring optimisation results from the four optimised patients to the two test patients during leave-pair-out, the threshold was set as the median plus range of the maximum metric value observed in the normal deliveries for the four patients. The metrics were evaluated at control points 19–180 in the normal deliveries, the maximum value of the metric was found, and the median and range of these maxima over the four patients were found. The value of the median plus range was taken to be the threshold. A distribution of maximum values was therefore implicitly assumed for the error-free cases, and the median plus range was taken to be the upper extreme of that distribution.

The optimisation process itself consisted of an exhaustive search over the combinations of *w_i_*, with allowed values ranging from 0.00 to 1.00 in steps of 0.05. In contrast with the individual metrics, the thresholds for the CDM varied according the weighting factors used to calculate the CDM. This was simply because the value of the CDM changed according to the weighting factors used. The threshold value was therefore recalculated every time that the weighting factors were updated, by taking the median plus range of the maximum error value in the normal deliveries for the four patients. The search method investigated each of the four patients in turn, and within each patient, the error cases in turn. The method examined each running sum of 10 control points in turn and evaluated both the individual metrics and composite metric. The index of the first segment at which an error was detected by any of these five metrics passing the threshold was noted and the objective value was calculated as the mean of these indices over the four patients and various error cases. The final objective value was therefore the minimum value of the mean segment index for all patients and error cases at which the errors could be detected.

### Evaluation of detection ability

**Figure 4.**
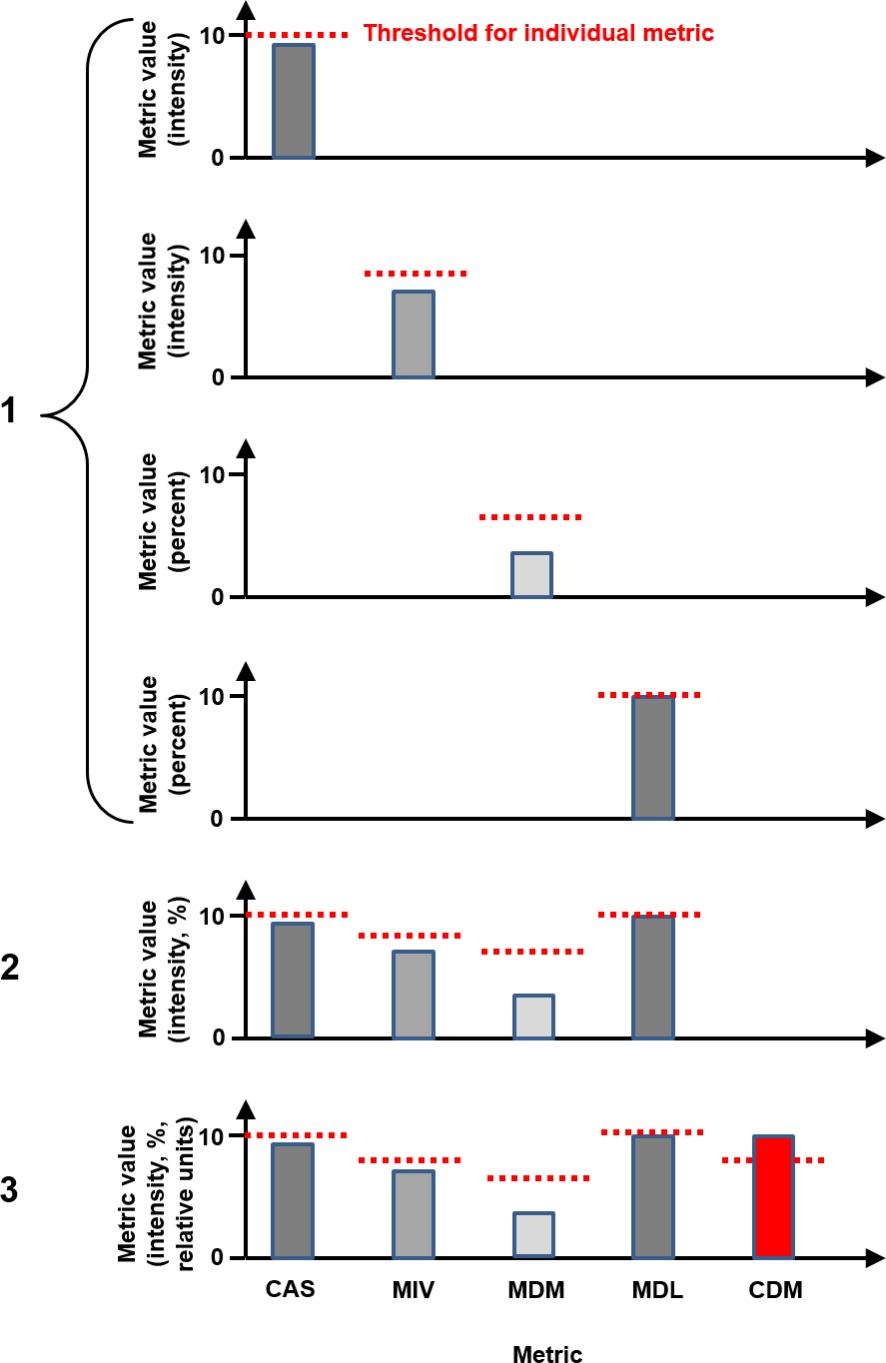
Three paradigms considered in this study. Each of the metrics is considered individually, then together, then in combination with the composite difference metric. The scales shown on the vertical axes are realistic, but the metrics and their thresholds are schematic only. CAS, central axis signal; CDM, composite difference metric; MDL, mean difference as a percentage of local intensity; MDM, mean difference as a percentage of maximum intensity; MIV, mean image value.

The thresholds for the individual metrics and the CDM, together with the weights of the CDM, were then applied to the two evaluation patients. For comparison purposes, the maximum values of the metrics, based on the section images (*i.e.* running sums of 10 control points), reached over segments 19–180, were evaluated. The mean index for the two patients at which an error was detected was also taken to represent the effectiveness of the metric in identifying an error. Specifically, three different error detection scenarios were evaluated (Figure 4):

1. Each of the four individual metrics was considered in isolation. For each metric in turn, the threshold was set and the mean index at which the errors were detected was noted.
2. The four individual metrics were considered separately but simultaneously. This corresponded most closely to what is currently carried out clinically. The threshold was determined for each individual metric, and then the first index at which *any* of the four metrics exceeded its respective threshold was noted.
3. The CDM was used in addition to the four individual metrics separately, *i.e*. in addition to Scenario 2. This scenario represented the results of the optimisation process.

## Results

**Figure 5.**
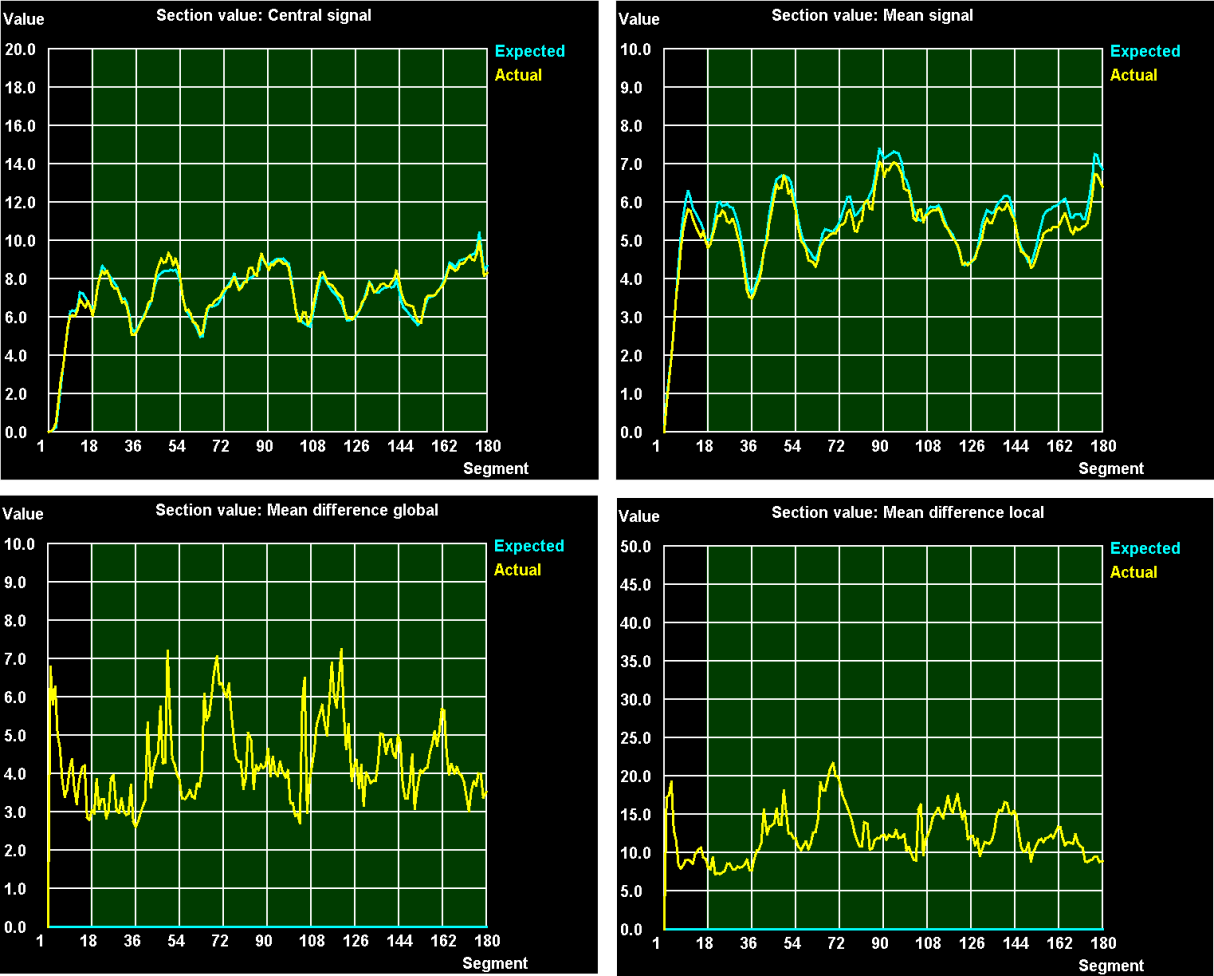
Section values of the four difference metrics for error-free delivery in one patient. The green background indicates that the error threshold has not been exceeded, and in the real-time implementation, this background changes to orange (near miss) or red (error) for those segments that exceed the chosen thresholds. Note that the scales differ on the vertical axes.

For all error-free treatment plans, the dose measured using a 0.6 cm^3^ ionisation chamber at the centre of the water-equivalent phantom is within 1% of the volume-averaged dose predicted by the AutoBeam inverse treatment planning system. Examples of the section values (*i.e.* running average of 10 segments) of the four individual metrics are shown in Figure 5 for the error-free case.

**Figure 6.**
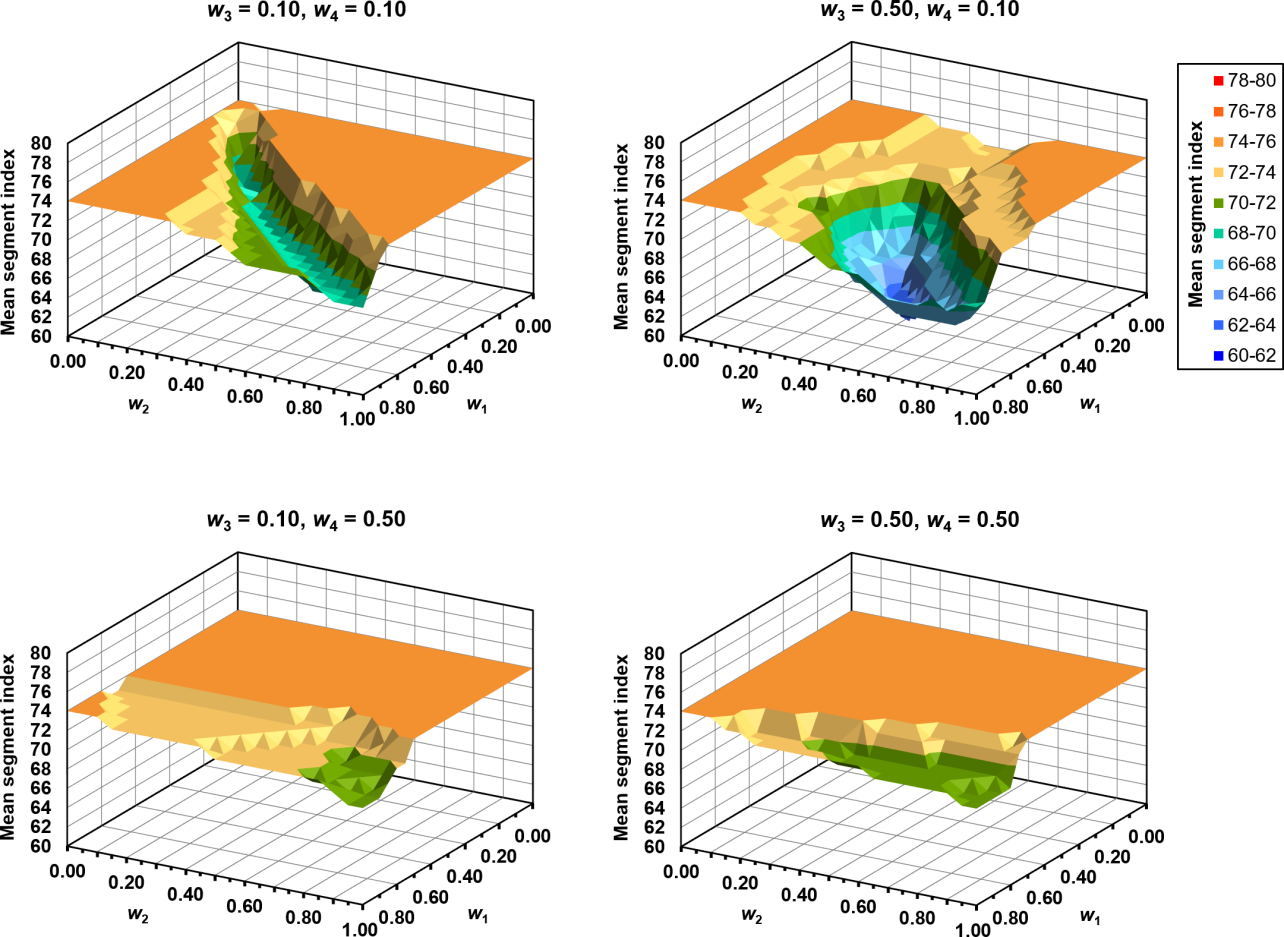
Mean segment index at which errors are first identified, as a function of the weighting factors, *w*.

The complete set of weighting factors searched by the optimisation method for the CDM constitutes a four-dimensional space, which is difficult to visualise in its entirety, but several planes through this space are shown in Figure 6. Each plot shows the variation of the mean segment index at which errors are first identified, which is the objective function value used by the weight optimisation. In each plot, the mean segment index is shown as a function of *w*_1_, the weight given to the central axis signal, and *w*_2_, the weight given to the mean image value. The four plots show this region for four different combinations of *w*_3_, mean difference relative to maximum image intensity, and *w*_4_, mean difference relative to local image intensity. For simplicity, this result does not use the leave-pair-out strategy, so all six patients are included. The value of 74 at which the segment index plateaus is the value attained by the individual metrics, so the combination of the individual metrics and the CDM always achieves a lower value than this.

At low values of *w*_3_ and *w*_4_, there is a clear minimum in the value of the segment index, reached with approximately equal values of *w*_1_ and *w*_2_. This minimum region rotates somewhat at higher values of *w*_3_ and *w*_4_, and is less marked. At values of *w*_3_ and *w*_4_ approaching unity, the segment index levels out at the plateau value of 74.

**Figure 7.**
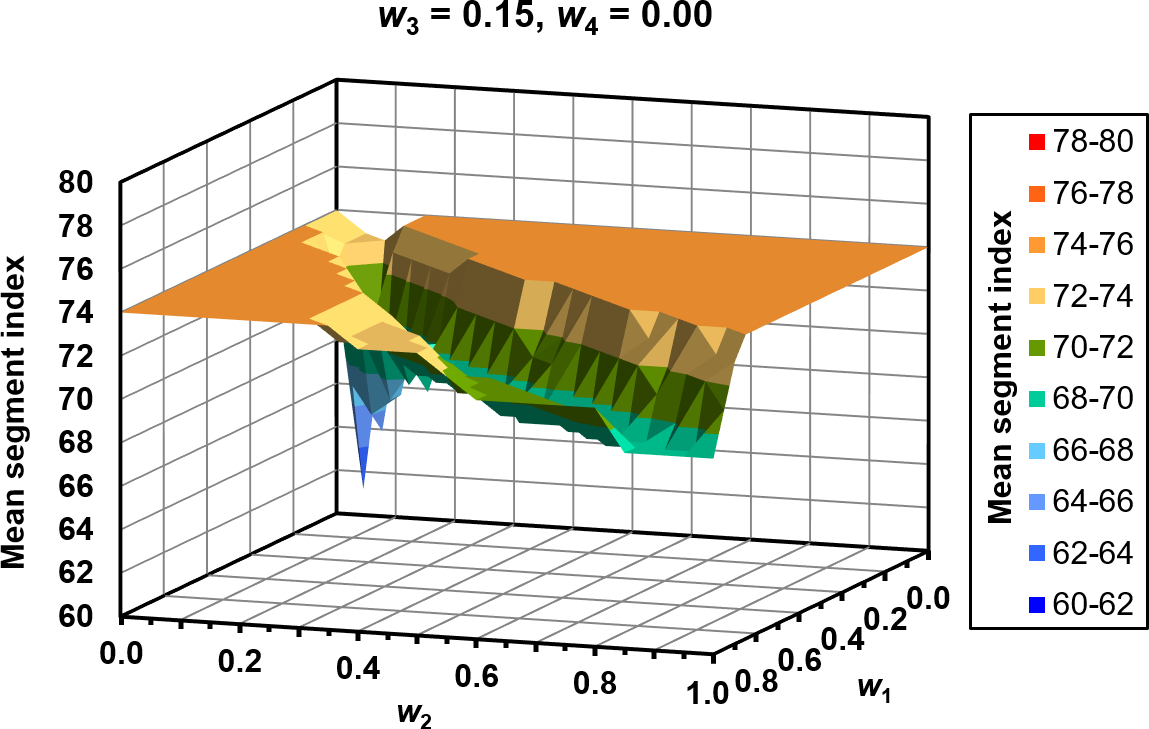
Mean segment index at which errors are first identified, as a function of the weighting factors, *w*, for the optimal combination of *w*_3_ and *w*_4_.

The median and range of the CDM weights chosen by the optimisation process are shown in Table 1. The constituent metrics are similarly weighted, with the exception of the mean difference relative to local image intensity. The form of the optimisation space for this exact combination of *w*_3_ and *w*_4_ is shown in Figure 7.

**Table 1.** Median and range values of the optimal weightings for the composite difference metric, obtained using the leave-pair-out method

Metric	Weight
CAS	0.15 (0.00–0.45)
MIV	0.10 (0.10–0.35)
MDM	0.15 (0.10–0.25)
MDL	0.00 (0.00–0.20)

CAS, central axis signal; MDL, mean absolute difference relative to local image intensity; MDM, mean absolute difference relative to image maximum; MIV, mean image value.

The segment index at which the errors are identified is summarised in Table 2. Each of the nine combinations of patient in the leave-pair-out approach yields a mean segment index for error identification, and the table shows the median and range of these values. Considering the metrics individually, the central axis signal is moderately sensitive to errors and allows timely detection of errors. Mean image value is not responsive to errors, because an increase in image intensity in one part of the image can be offset by a decrease in image intensity elsewhere. The mean absolute differences are the most effective ways of detecting errors in a timely manner, if a single metric is to be used. One patient of the six exhibits a false-positive result for the central axis signal and mean image value in all of the three cases where that patient is used in the test set. In other words, the thresholds for those image metrics are exceeded for the normal delivery.

**Table 2.** Median and range values of the mean segment index at which errors are detected, using individual metrics, multiple separate metrics and the composite difference metric

Metric	Segment index
CAS	136 (58–170)
MIV	156 (138–181)
MDM	101 (82–146)
MDL	99 (85–141)
All of the above with respect to their own respective thresholds	87 (40–130)
All of the above plus CDM	73 (35–95)

CAS, central axis signal; CDM, composite difference metric; MDL, mean absolute difference relative to local image intensity; MDM, mean absolute difference relative to image maximum; MIV, mean image value.

Taking all of these metrics and examining them with respect to their respective thresholds gives a more timely response to errors than using any single metric. This is also shown in Table 2. A further reduction in mean value of segment index at which errors are detected is seen when the composite difference metric is used in addition to the individual metrics. The benefit of the CDM, expressed as a ratio of the segment index for error detection using multiple separate metrics plus CDM *vs* that using only multiple separate metrics, has a median value of 0.83 (range 0.73–0.98). The one patient that exhibits a false-positive result for the individual metrics also shows a false-positive result for multiple separate metrics and for the CDM, since these metrics are based on the individual metrics (see paradigms 2 and 3 of Figure 4).

**Figure 8.**
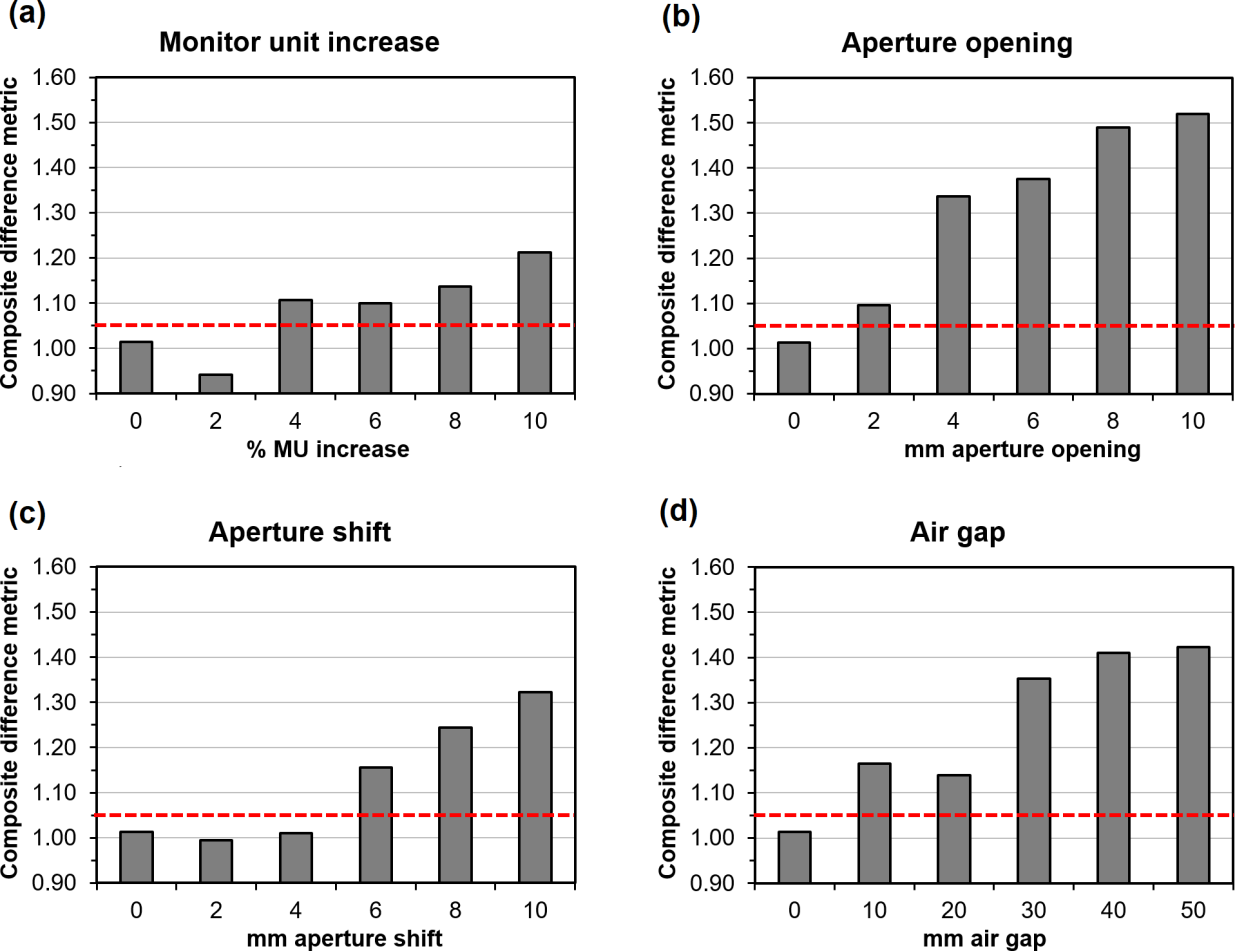
Maximum value attained by the composite difference metric in a single patient over segments 19–180 for various error types and magnitudes. The dashed line shows the threshold, based on the case with no error.

**Figure 9.**
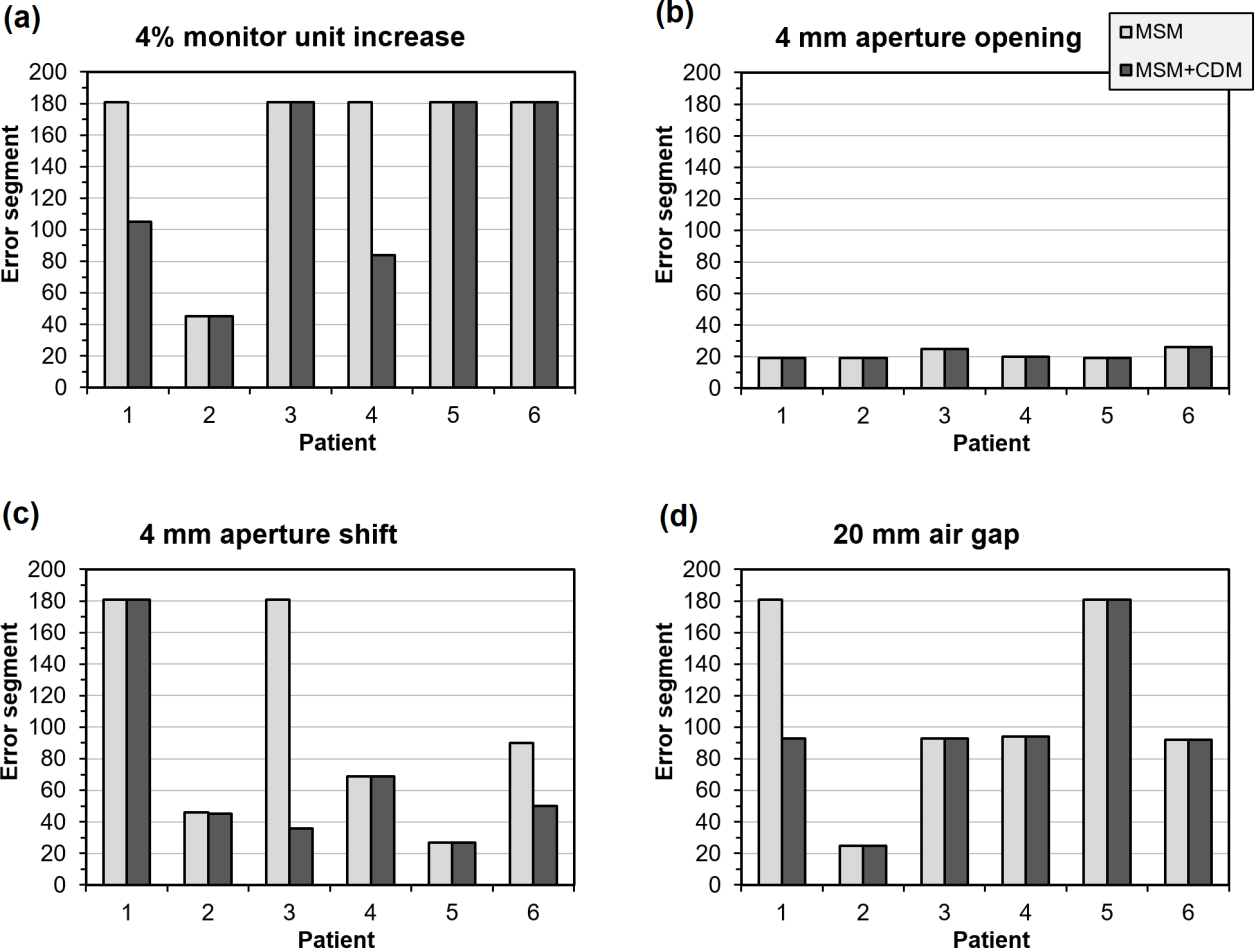
First segment at which an error is detected in the six patients, for various error types. CDM, composite difference metric; MSM, multiple separate metrics.

These results are based on multiple patients, various types of error and various magnitudes of error taken together, but the magnitudes of the composite difference metric for the various errors separately are shown for a single patient in Figure 8. This figure shows the maximum value of the CDM attained in segments 19–180. The indices of the segments at which the errors are first detected, by passing the thresholds, are shown for a single error magnitude in all patients in Figure 9. Note that the values of the individual metrics are not explicitly included in Figure 8, but they are used both individually and in the CDM in Figure 9. From Figure 9, it can be seen that an aperture opening is the earliest to be detected of the four types of error, followed by an aperture shift, with a monitor unit error and air gap error being less easily detected. It is clear that the inclusion of the CDM increases the responsiveness of the system to errors. For the 4 mm aperture opening, there is little difference between multiple separate metrics and use of the CDM due to the immediate detection of errors at Segment 19 in both instances. There is also very little difference in the case of a 4 mm aperture shift, for a similar reason. However, in certain cases, there are very marked improvements in error detection.

## Discussion

The composite difference metric used in this study shows improved timeliness of error detection compared to a single difference metric or multiple difference metrics compared against their own respective thresholds. The mathematical form of the CDM itself gives maximum benefit when several individual metrics show slightly elevated values. Other forms may be possible, but empirical experimentation has shown that forms such as a simple-weighted sum or weighted product are not as effective as the form given by Equation (1), and in some cases fail to produce any benefit at all. Nevertheless, the metrics used in this study are presented as a proof of concept, and there is scope for further investigation of different forms of metric. With this in view, the optimisation method presented in this study (Figure 3) is not dependent on the exact form of the CDM used, as the CDM is merely evaluated at each iteration of the process. The magnitude of the improvement with the CDM is around 15–20%, which is expected to translate into a corresponding sparing of incorrectly delivered dose, should an error occur.

Unlike the specific tests used by Passarge et al,^31^ each of which is designed to identify a particular error, the metrics used in the present study are general image comparison metrics. This is to enable as broad as possible a range of errors to be identified. Furthermore, it is likely that the same approach can be used in the context of back-projection portal dosimetry, but with the metrics chosen as structure-based, dosimetric metrics. For example, Olaciregui-Ruiz et al^22^ use a variety of dosimetric statistics in conjunction with the back-projection method to identify anatomical changes during treatment. Similarly, the optimal weightings of the composite difference metric may vary for different treatment sites, in the same way that alert criteria may be adapted specifically for each treatment site.^32^ γ analysis has not been used in this study as allowing a small spatial error in the segments of a VMAT plan may cause too large an error in the overall delivered dose.

As Mijnheer et al^33^ point out, the power to detect errors depends on the baseline accuracy of the portal dosimetry prediction method. If there is a large difference between predicted and measured images in the absence of errors, the thresholds are high, so that errors are less quickly detected. With this in mind, care has been taken in this study to ensure as accurate a prediction model as possible, with the inclusion of couch attenuation and gantry sag in the model. The images are initially collated according to segment of the treatment plan and then a running sum of 10 segments is used to give an optimum balance between agreement with predictions and useful information on sections of the arc. Similar considerations are also used by other authors.^34,35^

Even with careful attention to the quality of the prediction and the handling of the images, there is still some dependence on the thresholds used to signify an error. In clinical practice, the thresholds are set from a sample of normal patient results, and then applied to further patients. This has been modelled in this study by calculating the thresholds on the optimisation patients and then applying them to the evaluation patients. By estimating the maximum metric value likely to be encountered in the normal deliveries using the distribution of maxima in the optimisation patients, the number of false positives is minimised. However, one patient in this study, with higher metric values for the normal delivery, gives false-positive results. False positives are particularly unwanted in the real-time context as they are disruptive to the treatment of the patient. It is clear that a much larger collection of normal data are needed for the establishment of optimal thresholds, so that errors can be detected quickly, but false positives do not occur frequently.

This observation suggests that the use of artificial neural networks and deep learning may be beneficial for combining the metrics without requiring explicit thresholds.^36–38^ In this case, the thresholds are implicitly built into the neural network in the various nodes and layers from which the network is composed. The connection together of the initial nodes of the network by the deeper layers may provide a similar effect to the composite difference metric examined in the present study.

As with other studies,^26,33,39^ the method more readily identifies errors in MLC leaf positioning and is less accurate in identifying material changes such as air gap errors (Figure 9). The sensitivity to MLC opening errors occurs because there is an increased output factor as well as a shift in position. Consequently, this type of error can be identified quickly in the sequence of segments. This study does not investigate patient positioning errors as portal dosimetry is not very sensitive to positioning errors,^5,33^ and is therefore best implemented in conjunction with some means of position verification such as cone beam CT. There are indications, however, that the segment-resolved nature of real-time portal dosimetry does allow for some detection of these errors.^21^

## Conclusions

A composite difference metric constructed from a weighted power sum of individual metrics is a valuable addition to the process of error detection in the context of intrafraction segment-resolved portal dosimetry. The metric is able to identify variations in measured portal images with respect to predicted images before the separate measures exceed their respective tolerances. Optimal values of the weighting factors for the metric can be found by using an exhaustive search in conjunction with appropriate thresholds based on normal deliveries. Application of the method to a series of prostate treatment images shows that simulated errors can be detected approximately 20% earlier than when using several measures separately.
